# Isolation and Characterization of Plant Growth-Promoting Endophytic Bacteria *Paenibacillus polymyxa* SK1 from *Lilium lancifolium*

**DOI:** 10.1155/2020/8650957

**Published:** 2020-02-27

**Authors:** Mohammad Sayyar Khan, Junlian Gao, Xuqing Chen, Mingfang Zhang, Fengping Yang, Yunpeng Du, The Su Moe, Iqbal Munir, Jing Xue, Xiuhai Zhang

**Affiliations:** ^1^Beijing Agro-Biotechnology Research Center, Beijing Academy of Agriculture and Forestry Sciences, Beijing 100097, China; ^2^Genomics and Bioinformatics Division, Institute of Biotechnology and Genetic Engineering (IBGE), The University of Agriculture, Peshawar 25000, Khyber Pakhtunkhwa, Pakistan; ^3^Pharmaceutical Research Laboratory, Biotechnology Research Department, Ministry of Education, Mandalay Division, Kyaukse 05151, Myanmar

## Abstract

*Paenibacillus polymyxa* is a plant growth-promoting rhizobacterium that has immense potential to be used as an environmentally friendly replacement of chemical fertilizers and pesticides. In the present study, *Paenibacillus polymyxa* SK1 was isolated from bulbs of *Lilium lancifolium*. The isolated endophytic strain showed antifungal activities against important plant pathogens like *Botryosphaeria dothidea*, *Fusarium oxysporum*, *Botrytis cinerea*, and *Fusarium fujikuroi*. The highest percentage of growth inhibition, i.e., 66.67 ± 2.23%, was observed for SK1 against *Botryosphaeria dothidea* followed by 61.19 ± 3.12%, 60.71 ± 3.53%, and 55.54 ± 2.89% against *Botrytis cinerea*, *Fusarium fujikuroi*, and *Fusarium oxysporum*, respectively. The metabolite profiling of ethyl acetate fraction was assessed through the UHPLC-LTQ-IT-MS/MS analysis, and putative identification was done with the aid of the GNPS molecular networking workflow. A total of 29 compounds were putatively identified which included dipeptides, tripeptides, cyclopeptides (cyclo-(Leu-Leu), cyclo(Pro-Phe)), 2-heptyl-3-hydroxy 4-quinolone, 6-oxocativic acid, anhydrobrazilic acid, 1-(5-methoxy-1H-indol-3-yl)-2-piperidin-1-ylethane-1,2-dione, octadecenoic acid, pyochelin, 15-hydroxy-5Z,8Z,11Z, 13E-eicosatetraenoic acid, (Z)-7-[(2R,3S)-3-[(2Z,5E)-Undeca-2,5-dienyl]oxiran-2-yl]hept-5-enoic acid, arginylasparagine, cholic acid, sphinganine, elaidic acid, gossypin, L-carnosine, tetrodotoxin, and ursodiol. The high antifungal activity of SK1 might be attributed to the presence of these bioactive compounds. The isolated strain SK1 showed plant growth-promoting traits such as the production of organic acids, ACC deaminase, indole-3-acetic acid (IAA), siderophores, nitrogen fixation, and phosphate solubilization. IAA production was strongly correlated with the application of exogenous tryptophan concentrations in the medium. Furthermore, inoculation of SK1 enhanced plant growth of two *Lilium* varieties, Tresor and White Heaven, under greenhouse condition. In the light of these findings, the *P. polymyxa* SK1 may be utilized as a source of plant growth promotion and disease control in sustainable agriculture.

## 1. Introduction

The plant growth-promoting rhizobacteria (PGPR) and endophytes have been the prime focus of research on increasing plant's ability to grow better in changing environmental biotic and abiotic conditions. These PGPR increase plant growth and productivity directly or indirectly through several mechanisms, including synthesis of plant growth hormones, biological nitrogen fixation and phosphate solubilization [1, 2], siderophores and organic acid production, and plant tolerance to abiotic stress through lowering host ethylene levels by 1-aminocyclopropane-1-carboxylate (ACC) deaminase activity [3, 4]. Several of these PGPR confer biotic stress tolerance to plants through direct or indirect mechanisms by blocking the rhizosphere colonization by pathogenic and parasitic organisms. Secretion of antagonistic compounds and lysis of pathogens cell walls are used as direct mechanisms of pathogens inhibition [5, 6]. Indirect mechanisms may involve the induction of plant defense pathways, altered production of stress-related proteins and other compounds [7], and competition for essential nutrients, limited in the soil [8, 9].

Members of the genus *Paenibacillus* are Gram-positive, sporulating, and facultative anaerobes. The genus contains more than 150 species and most of them play important roles as PGPR in agriculture [10, 11]. Several plant-associated members of *Paenibacillus* improve plant growth and productivity through the production of indole acetic acid (IAA) and other phytohormones, phosphate solubilization, and atmospheric nitrogen fixation by some species [12].

*Paenibacillus polymyxa* is an important member of the genus which was previously characterized as *Bacillus polymyxa* [13]. Different strains of *P. polymyxa* have been reported as increasing plant fitness and disease resistance through the secretion of plant growth hormones, antimicrobial and volatile compounds, nutrient cycling, and pathogen antagonism [10, 14, 15]. *P. polymyxa* plays an important role in plant protection against pathogenic fungi, oomycetes, and bacteria mainly by the production of antibiotic compounds such as polymyxins and fusaricidins [16]. Due to these properties, the *P. polymyxa* strains have gained much research attention as important players in biofertilization, biocontrol, and biofuel applications [12].

The genus *Lilium* is comprised of herbaceous flowering plants growing from bulbs. The various *Lilium* species are widely cultivated in eastern countries for cut flowers and used in the food and medicine industries. Since ancient times, people in eastern Asia have been using *Lilium* bulbs as medicinal and culinary herbs [17]. The medicinal uses of *Lilium* species are evident from findings of several research studies where the bulb extracts showed antimicrobial, antivirus, and anti-inflammatory activities [18–20]. Due to these pharmacological, food, and cut flower demands of the *Lilium* species, research has been focused to improve plant growth and bulbs yield under pathogenic diseases and abnormal climatic conditions. PGPR isolation and plant inoculation may be one of the attractive approaches for increasing plant growth and the productivity of *Lilium* bulbs. The *Lilium* plant-associated rhizosphere and endophytic microorganisms may be isolated to increase plant growth and bulbs yield upon inoculation. The current study aimed to identify endophytic bacteria from bulbs of *Lilium lancifolium* and characterize their antimicrobial, plant growth-promoting (PGP), secondary metabolites, and plant inoculation properties.

## 2. Materials and Methods

### 2.1. Sample Collection

Bulbs of *Lilium lancifolium* were collected from the experimental fields in December 2018 and were brought to the Laboratory of Beijing Agriculture Biotechnology Research Center, Academy of Agriculture and Forestry Sciences, China. Fresh and healthy-looking bulbs were selected for endophytes isolation.

### 2.2. Sample Sterilization and Endophytes Isolation

Samples' preparation and isolation of endophytes were carried out using a previously described method [21]. Bulbs were first washed with tap water to remove any dust particles. The outermost layers of the bulbs were peeled off and the inner portions were washed with tap water for 5 min. Samples were then immersed in 70% (v/v) ethanol for 1 min followed by immersion in 10% (concentration of active chlorine) NaClO solution for 20 min. The bulbs were then washed with sterile distilled water. After surface sterilization, the outer layer on both sides of each bulb portion was removed. The bulb portions were then cut aseptically into approximately 1 cm × 1 cm pieces and inoculated on LB agar plates. The plates were incubated at 30 ± 1°C until bacterial growth started on the bulb portions. After 2-3 days' incubation, the individual bacterial colonies were aseptically inoculated into fresh LB broth and incubated at 30 ± 1°C until pure cultures were obtained by serial subculturing. The isolated endophytes were stored as glycerol stocks at −80°C.

### 2.3. Identification of Bacterial Strain

The bacterial strain was cultured on LB agar (yeast extract 5 g·L^−1^, tryptone 10 g·L^−1^, NaCl 10 g·L^−1^, agar 15 g·L^−1^, pH 7.0–7.5) and incubated at 30°C for 24 h. The bacterial strain was characterized using morphology, growth pattern, Gram staining, and electron microscopy. The Gram reaction was performed as previously described [22]. For scanning electron microscopic observations, bacterial cells were cultured in LB for 12 h at 30°C. About 1 ml LB culture was centrifuged at 8000 rpm for 5 min. The supernatant was discarded and the cell pellet was washed thrice with 1 ml 0.2 M phosphate buffer (PBS) (pH 7.2–7.4). The pellet was then fixed with 2.5% glutaraldehyde for 3 h. After fixation, the pellet was washed twice with PBS followed by rinsing with pure water. The pellet was then dehydrated by the concentration gradient of 30%, 50%, 70%, 80%, and 90% of ethanol for 15 min at each step and then dehydrated twice for 15 min in 100% ethanol. Cell morphology was determined using SU8010 field-emission scanning electron microscope (SEM, Hitachi, Japan). The accelerating voltage was 5 kV, and images were collected digitally from the emitted secondary electron signal.

For molecular analysis, the endophytic strain was inoculated in LB broth at 30°C in a shaker at 220 rpm. The overnight culture was centrifuged at 4000 rpm, room temperature for 10 min. The supernatant was discarded and the cell pellet was used for genomic DNA extraction using the Bacterial Genomic DNA Isolation Kit (SolarBio) in accordance with the manufacturer's protocols. The isolated endophyte was identified by the sequences of 16S ribosomal RNA (rRNA) genes. About 1500 bp sequence was amplified from genomic DNA using primers P027F and 1378R specific for the 16S ribosomal RNA genes. A 25 *μ*l PCR reaction contained 1 *μ*l (0.5–10.0 ng) of template DNA, 0.2 *μ*M of primers P027F (5′-GAGAGTTTGATCCTGGCTAG-3) and 1378R (5′-CGGTGTGTACSSGGCCCGGGAACG-3′) each, 200 *μ*M of each dNTP, 10X buffer, 2 mM MgSO_4_, and 1 U High-Fidelity KOD Taq DNA Polymerase. The cycle parameters were as follows: initial denaturation at 94°C for 4 min; 30 cycles of denaturation for 30 s at 94°C, annealing for 1 min at 63°C, and extension for 1 min at 68°C; and a final overall extension for 7 min at 68°C. The PCR product was purified using the QIAquick PCR Purification Kit (Qiagen, Hilden, Germany) and was then sequenced through Beijing Biomed Gene Technology Co. Ltd. Sequences were BLAST searched against homologous bacterial 16S ribosomal RNA sequences through NCBI. The determined sequences were aligned using CLUSTAL W, and phylogenetic tree was constructed based on the maximum likelihood (ML) algorithm using the MEGA 7 software [23]. The nucleotide sequence was then submitted to GenBank under accession number MN 326755.1.

### 2.4. Antifungal Activity

Antifungal activities of the isolated endophyte SK1 were tested under *in vitro* conditions against four strains of pathogenic fungi, i.e., *Botryosphaeria dothidea*, *Fusarium oxysporum*, *Botrytis cinerea*, and *Fusarium fujikuroi*. The antifungal bioassays were conducted based on a dual culture method [24]. The two-day-old bacterial cultures were used against the pathogenic fungal strains. About 10 *μ*l of the bacterial culture was spot-inoculated at four corners of the PDA plate, approximately 2.5 cm away from the center. A fungal plug of 6 mm was placed at the center of the plate. The plates were incubated at 28°C. Plates containing the fungal plugs without bacterial inoculation were used as controls. Plates were checked regularly for the growth of the fungal pathogen against the endophytic bacterial strain. The zone of inhibition of fungal growth was measured after the fungal mycelia in the control plates reached the edges of the plates. Growth inhibition of the fungal pathogen was calculated using the following formula: % of growth inhibition = [(*C* − *T*)/*C*] × 100, where *C* is the radial growth of the test pathogen in the control plates (mm), and *T* is the radial growth of the test pathogen in the test plates (mm). The experiment was repeated thrice.

### 2.5. Ethyl Acetate Extraction of Secondary Metabolites

The extraction of secondary metabolites of the SK1 strain was done by solvent partition method. The strain was grown in LB broth at 30°C and 150 rpm shaking for 5-6 days. After incubation, the broth cultures were centrifuged at 10000 rpm, 4°C for 10 min. The supernatant was filtered through a 0.2 *μ*m syringe filter. An equal volume of the filtrate and ethyl acetate was taken into the separating funnel and shaken for complete extraction. The solvent phase that contained secondary metabolites was separated from the aqueous phase, and the solvent was evaporated to dryness to yield the crude extracts. The crude extract, about 20 mg, was redissolved in 1 ml of 70% methanol. 500 *μ*l of the dissolved extract was filtered through a 0.2 *μ*m syringe filter before ultrahigh-performance liquid chromatography LTQ XL linear ion trap mass spectrometry/mass spectrometry (UHPLC-LTQ-XL-IT-MS/MS) analysis.

### 2.6. UHPLC-LTQ-XL-IT-MS/MS Analysis for Secondary Metabolite Profiling

UHPLC-LTQ-IT-MS/MS analysis was performed using the method partially adapted from Lee et al. [25]. The Thermo Fisher Scientific LTQ XL linear ion trap mass spectrometry consisted of an electrospray interface (Thermo Fisher Scientific, San José, CA, USA) coupled with a DIONEX UltiMate 3000 RS Pump, RS Autosampler, RS Column Compartment (Dionex Corporation, Sunnyvale, CA, USA) used for secondary metabolite profiling of the extract. The sample was separated on a Thermo Scientific Hypersil GOLD C18 column with 1.9 *μ*m particle size. The mobile phase consisted of A (0.1% (v/v) formic acid in water) and B (0.1% (v/v) formic acid in acetonitrile), and the gradient conditions were increased from 10% to 100% of solvent B. Scanning was set to start after 1 min to source. Solvent gradient time was set over 19 min and reequilibrated to the initial condition for 4 min by setting the divert valve to waste. The flow rate was set at 0.3 ml/min and the injection volume was 10 *μ*l. The temperature of the column during measurement was maintained at 35°C. The ion trap was performed in positive and full-scan ion modes within a range of 150–1000 *m*/*z*. The operating parameters were as follows: source voltage, ±5 kV; capillary voltage, 39 V; capillary temperature, 275°C; auxiliary gas flow rate, 10−20 arbitrary units; sheath gas flow rate, 40−50 arbitrary units; and spray voltage, 4.5 kV. Tandem MS (MS/MS) analysis was performed by scan-type turbo data-dependent scanning (DDS) under the same conditions used for MS scanning for the six most intense ions using the Nth order double play mode. MS data was acquired by Xcalibur software, Thermo Fisher Scientific.

### 2.7. Putative Identification of Secondary Metabolites

Putative identification of secondary metabolites was done using molecular networking workflow from the GNPS website (https://gnps.ucsd.edu) [26]. Raw LC-MS file was converted into mzXML using ProteoWizard 3.0.19140 [27], and the mzXML file was uploaded to GNPS. A molecular network was created using the default parameters. The spectra in the network were then searched against GNPS spectral libraries. The library spectra were filtered in the same manner as the input data. All matches kept between network spectra and library spectra were required to have a score above 0.7 and at least 6 matched peaks.

### 2.8. Plant Growth-Promoting (PGP) Assays

Several qualitative and quantitative tests were conducted to determine important plant growth-promoting traits. These included the detection of organic acids, indole acetic acid (IAA), 1-aminocyclopropane-1-carboxylate (ACC) deaminase, siderophores, phosphate solubilization, and nitrogenase activity. For PGP assays, the bacterial strain was cultured in 1 ml LB media for 48 h at 30°C with shaking. The culture was then centrifuged at 4000 rpm for 10 min at room temperature. The supernatant was removed and the cell pellet was washed two times with 1 ml of MgSO_4_ (10 mM) and resuspended in 650 *μ*l of MgSO_4_. This cell suspension was then used for PGP assays.

### 2.9. Organic Acid Production Assay

Organic acids were detected according to the previously developed protocol [28]. About 50 *μ*l of the bacterial suspension in MgSO_4_ (10 mM) was inoculated in 800 *μ*l of sucrose tryptone medium (ST) containing sucrose (20 g·L^−1^) and tryptone (5 g·L^−1^). The ST medium was supplemented with 10 ml of trace elements solution. Trace element solution contained CuSO_4_·5H_2_O (20 mg·L^−1^), FeCl_3_ (100 mg·L^−1^), H_3_BO_3_ (20 mg·L^−1^), NaMoO_4_ (20 mg·L^−1^), MnCl_2_·4H_2_O (20 mg·L^−1^), and ZnCl_2_ (280 mg·L^−1^). Samples were incubated for 5 days at 30°C and 200 rpm. Organic acids were detected by adding 100 *μ*l of 0.1% alizarin red S pH indicator to all samples. After 15 min, samples showing the color change to yellow were considered as positive, while pink indicated negative results.

### 2.10. Indole Acetic Acid (IAA) Detection

Indole acetic acid (IAA) was detected according to the method of Gordon and Weber [29] with minor modifications. A bacterial suspension of 150 *μ*l in 10 mM MgSO_4_ was inoculated in 3 ml of 1/10 diluted 869-rich medium. The medium was supplemented with four different concentrations of tryptophan, i.e., 0 mg·ml^−1^, 2 mg·ml^−1^, 4 mg·ml^−1^, and 6 mg·ml^−1^. Samples were incubated at 30°C for 4 days. After incubation, the bacterial cultures were centrifuged at 4000 rpm for 20 min and 1 ml of the supernatant was mixed with 2 ml of Salkowski's reagent (98 ml 35% HClO_4_, 2 ml 0.5 M FeCl_3_). After 20 min, the development of pink color was considered as positive for IAA production. Indole acetic acid was further quantified by measuring OD at 530 nm in a spectrophotometer. The IAA quantities in samples were measured based on a standard curve of known values ([Supplementary-material supplementary-material-1]).

### 2.11. ACC Deaminase Detection

The ability of the endophyte to produce 1-aminocyclopropane-1-carboxylate (ACC) deaminase was determined according to the previously developed method [30] with slight modifications [31]. About 250 *μ*l of the bacterial suspension in MgSO_4_ (10 mM) was added to 1.2 ml of salts minimal medium (SMN) containing 5 mM ACC as a sole source of N. The cultures were then incubated at 30°C for 3 days with shaking at 150 rpm. Samples were then centrifuged at 4000 rpm for 20 min at room temperature. The supernatant was removed and the pellets were resuspended in 100 *μ*l of Tris–HCl buffer (0.1 M) (pH = 8.5). Bacterial cells were disrupted by adding 3 *μ*l toluene followed by vigorous vortexing. Furthermore, 10 *μ*l of ACC (0.5 M) and 100 *μ*l of Tris–HCl buffer (0.1 M) (pH = 8.5) were added, and the samples were gently vortexed for 10 min. Samples were then incubated at 30°C for 30 min with shaking at 150 rpm. After incubation, 690 *μ*l of 0.56 N HCl and 150 *μ*l of 0.2% 2, 4-dinitrophenylhydrazine reagent (in 2 N HCl) were added to the cell suspensions. Samples were incubated at 30°C for 30 min, followed by the addition of 1 ml NaOH (2 N). Samples without the addition of ACC were used as negative controls. The color change from yellow to brown was considered as positive.

### 2.12. Siderophores Detection

The potential of isolated endophyte for siderophores production was evaluated through qualitative and quantitative tests. The bacterial cells were inoculated in liquid 284 medium with a chrome azurol S (CAS) shuttle solution, a method developed by Schwyn and Neilands [32]. The 284 medium with CAS solution stimulates siderophore production. About 50 *μ*l of the bacterial suspension in MgSO_4_ (10 mM) was inoculated in microcentrifuge tubes containing 800 *μ*l of 284 medium prepared with three different iron concentrations. The iron concentrations used were 0 *μ*M, 0.25 *μ*M, and 3 *μ*M Fe(III) citrate. Samples were incubated for 5 days at 30°C with shaking (150 rpm). After incubation, 100 *μ*l of the blue Chromium Azurol S (CAS) reagent was added to samples. Tubes were kept for 4 h at room temperature. Afterwards, the change of color from blue to orange/yellow was considered as positive. Siderophores concentrations in all samples were further measured at 630 nm. The siderophore quantities were measured as % of siderophore units by the formula: % of siderophore units = Ar − As/Ar ∗ 100, where “Ar” is the absorbance of reference (CAS reagent) and “As” is the absorbance of the sample at 630 nm. Siderophores production in bacterial isolates was further confirmed through a qualitative test using CAS agar assay. Briefly, the CAS solution with FeCl_3_ and HDTMA was added to Minimal Media 9 (MM9) containing 20% glucose, casamino acid solution, and bacto agar. Bacterial isolates were inoculated on CAS agar plates and incubated at 28°C under dark condition for two weeks. The appearance of yellow/orange hallows around the colonies confirmed siderophore production. All assays were carried out in triplicate.

### 2.13. Nitrogen Fixation Assay

A single colony of *P. polymyxa* strain SK1 and *Escherichia coli* O157 : H7 grown on solid LB medium was streaked onto solid nitrogen-deficient malate medium (NFM: 0.02 g·L^−1^ CaCl_2_, 0.1 g·L^−1^ NaCl, 0.01 g·L^−1^ FeCl_3_, 0.4 g·L^−1^ KH_2_PO_4_, 0.5 g·L^−1^ K_2_HPO_4_, 0.2 g·L^−1^ MgSO_4_·7H_2_O, 0.002 g·L^−1^ Na_2_MoO_4_·2H_2_O, 5 g·L^−1^ sodium malate, 15 g·L^−1^ agar, pH 7.2–7.4 using KOH) supplemented with 50 mg·L^−1^ yeast extract [33]. After incubation and colonies' appearance, a resulting single colony was then restreaked onto NFM to confirm the ability to fix nitrogen [34]. Plates were incubated at 28°C for 7 days.

### 2.14. Phosphate Solubilization Assay

Phosphate solubilization was determined according to the method previously described [35]. The *P. polymyxa* strain SK1 was cultured on solid NBRIP medium (1% glucose, 0.5% Ca_3_(PO_4_)_2_, 0.5% MgCl_2_, 0.01% (NH_4_)_2_SO_4_, 0.025% MgSO_4_.7H_2_O, 0.02% KCl, 1.5% agar), where growth is associated with the capacity to use inorganic phosphate in the form of Ca_3_(PO_4_)_2_ as a sole phosphate source. Plates were grown at 28°C for 14 days.

### 2.15. Experimental Design of Greenhouse Study

A greenhouse experiment was conducted to determine the plant growth-promoting effects of the isolated bacterial strain SK1 on selected *Lilium* varieties, Tresor and White Heaven, which are commercially cultivated in China. Normal and healthy-looking, same sized bulbs of both varieties were selected from the storage house at 4°C. Before inoculation, the overnight culture of SK1 in 5 ml LB was further inoculated in 50 ml LB and was cultured for 24 h at 30°C with 220 rpm shaking. Optical density (OD) of the overnight culture was determined and was then inoculated in 400 ml LB and was kept to grow for 24 h. This culture was then diluted 10 times with distilled water and bulbs of both varieties were soaked in the diluted culture for 40 min. The noninoculated bulbs of both varieties, soaked in simple LB, were used as controls. Soil pots of sizes 20 × 30 cm were prepared with a soil mix of peat moss, perlite, and vermiculite in a ratio of 2 : 1 : 1. Three bulbs of each variety, either inoculated or noninoculated (control), were sown in each soil pot. Pots were kept in a completely randomized design (CRD). Each treatment contained 5 pots. Pots were kept in plastic trays, which were watered with an equal amount of tap water with regular intervals. Morphological data such as plant height, number of flowering shoots, leaf length, leaf width, and bulbs weight was taken at the peak vegetative and reproductive stage.

### 2.16. Statistical Analysis

Data obtained from the greenhouse experiment was subjected to analysis of variance (ANOVA). Means were compared with Student's *t*-test at a probability of *α* = 0.05.

## 3. Results

**Figure 1:**
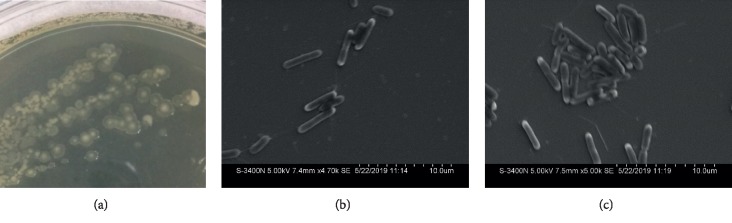
Colonies morphology and scanning electron microscope (SEM) analysis of the endophytic bacteria *P. polymyxa* SK1, isolated from *Lilium lancifolium.* The SK1 strain produced light pale, yellowish colored colonies on LB agar plates (a). The isolate is of rod-shaped structures (b) and (c).

**Figure 2:**
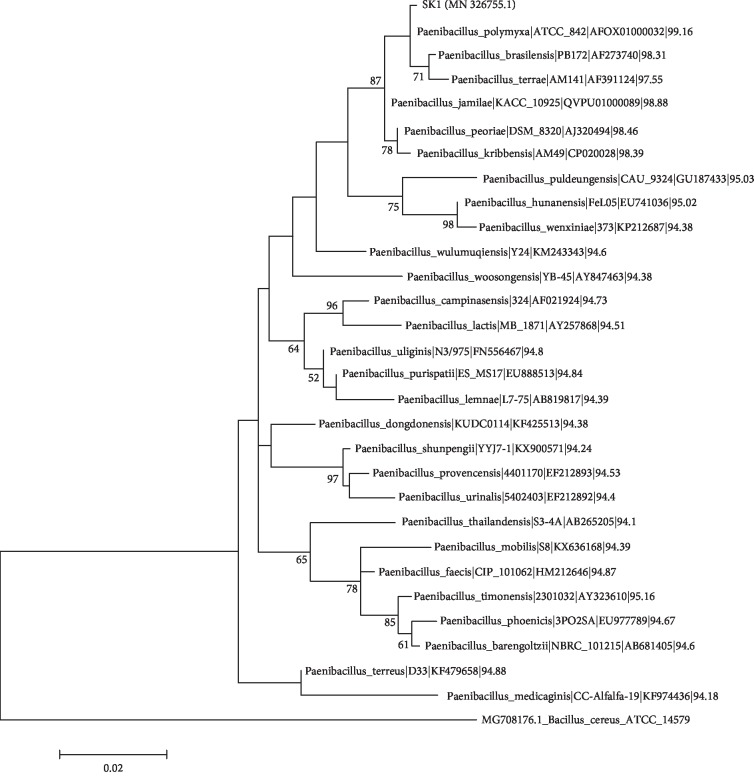
Phylogenetic analysis of 16S rRNA gene sequences of the bacterial endophyte SK1 isolated from *Lilium lancifolium* L. Sequences were aligned through “Clustal W” using MEGA 7 software. A phylogenetic tree was constructed using the maximum likelihood method. Bootstrap values are shown as percentages of 1000 replicates; values below 50% are not indicated. *Bacillus cereus* ATCC14579 (MG708176.1) was used as an outgroup sequence.

Several bacterial endophytes were isolated from the bulb samples of *Lilium lancifolium*. One isolate was identified as *Paenibacillus polymyxa* and was designated as SK1. The strain was further selected for the analysis of antifungal and plant growth-promoting effects. The SK1 strain formed light pale yellowish colonies with a thick central part surrounded by a light visible part on LB agar plates (Figure 1(a)). The isolate was a Gram-positive and spore-forming bacterium and exhibited small rod-shaped structures typical of the genus *Paenibacillus* as revealed by the scanning electron microscopic (SEM) analysis (Figures 1(b) and 1(c)). The BLAST results revealed that the 1427 bp long 16S rRNA gene sequence was closely related to *Paenibacillus polymyxa*. About 29 homologous 16S rRNA sequences including the query sequence of SK1 were aligned, and a phylogenetic tree was constructed using *Bacillus cereus* ATCC14579 (MG708176.1) as an outgroup sequence. Based on the maximum likelihood phylogenetic tree constructed with the 16S rRNA similarity (%), the SK1 strain showed 99.16% similarities with *P. polymyxa* strain ATCC842(T), (AFOX01000032) (Figure 2). The 16S rRNA gene sequence of the isolated SK1 strain shared high similarity with that of *Paenibacillus jamilae* (98.88%), *Paenibacillus peoriae* (98.46%), *Paenibacillus kribbensis* (98.39%), and *Paenibacillus brasilensis* (98.31%) The 16S rRNA gene sequence of the SK1 strain was submitted to GenBank, and the accession number was assigned as MN 326755.1.

**Figure 3:**
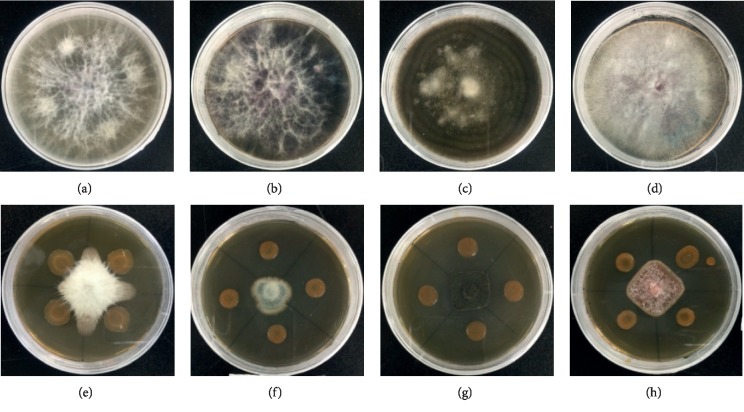
*In vitro* antifungal activities of the endophytic *P. polymyxa* SK1 against four fungal pathogens. A 5 mm fungus plug was inoculated into the center of the PDA medium surrounded by four spots of bacterial inoculum. Plates (a), (b), (c), and (d) are controls of *Fusarium oxysporum, Botryosphaeria dothidea, Botrytis cinerea*, and *Fusarium fujikuroi*, respectively. Plates (e), (f), (g), and (h) contain dual cultures of SK1 and the fungal pathogens. Antifungal activities were measured as theange of coloration from y size of the zones of inhibition of the pathogenic fungi. Zones of inhibitions were expressed as percentages.

The endophytic bacterial strain SK1 showed the high capability to inhibit mycelial growth of the test pathogens *Fusarium oxysporum*, *Botryosphaeria dothidea*, *Botrytis cinerea*, and *Fusarium fujikuroi* (Figure 3). Among these pathogenic fungi, *Fusarium fujikuroi* was first detected on the bulbs of *Lilium wardii* under *in vitro* conditions. This pathogenic strain was then identified through PCR amplification of ITS region and sequencing as *Fusarium fujikuroi*. These four pathogenic strains were used in an *in vitro* study showing the potential of causing infection in Tresor variety and *Lilium davidii* ([Supplementary-material supplementary-material-1]). The strain SK1 exhibited considerable inhibition potential against all the tested pathogenic strains, possibly due to the release of diffusible compounds against the test pathogens. Zones of inhibition of pathogenic fungi on PDA plates were measured as percentage values. The highest percentage of growth inhibition, i.e., 66.67 ± 2.23%, was observed for SK1 against *Botryosphaeria dothidea* followed by 61.19 ± 3.12%, 60.71 ± 3.53%, and 55.54 ± 2.89% against *Botrytis cinerea*, *Fusarium fujikuroi*, and *Fusarium oxysporum*, respectively (Table 1). These results suggested the highest activities for SK1 against the test pathogens.

**Figure 4:**
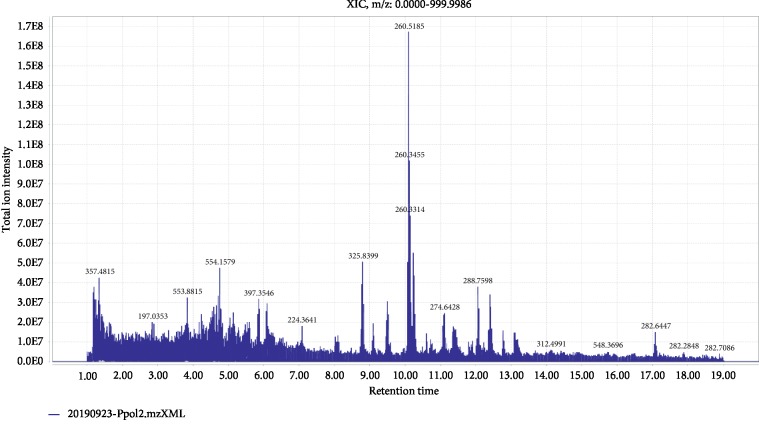
The total ion current (TIC) chromatogram of the ethyl acetate fraction of endophytic *P. polymyxa* SK1.

The metabolite profiling of ethyl acetate fraction was assessed through UHPLC-LTQ-IT-MS/MS analysis, and putative identification was done with the aid of the GNPS molecular networking workflow. Thermo raw files were converted into mzXML using ProteoWizard 3.0.19140, and mzXML files were uploaded to GNPS. The spectra in the network were then searched against GNPS spectral libraries. Tandem mass (MS/MS) spectra of some compounds in the ethyl acetate fraction of *P. polymyxa* SK1 were closely matched to the GNPS reference library (cosine score above 0.7 and at least 6 matched peaks) and were putatively identified as listed in Table 2. After removing the matched compounds from the media control, a total of 29 compounds were putatively identified, and the overview of their information related to molecular formula, *m/z* measured, library *m/z*, GNPS score, and GNPS library IDs can be seen in Table 2. The total ion chromatogram showing the intensities of all the detected peaks was given in Figure 4. The bioactive secondary compounds and metabolites identified in the ethyl acetate fraction of *P. polymyxa* SK1 included dipeptides, tripeptides, cyclopeptides (cyclo-(Leu-Leu), cyclo(Pro-Phe)), 2-heptyl-3-hydroxy 4-quinolone, 6-oxocativic acid, anhydrobrazilic acid, 1-(5-methoxy-1H-indol-3-yl)-2-piperidin-1-ylethane-1,2-dione,5-hydroxy-2-[2-hydroxy-3-[(2S,3R,4S,5S,6R)-3,4,5-trihydroxy-6-(hydroxyl methyl)oxan-2-yl]oxyphenyl]-7,8-dimethoxychromen-4-one, octadecenoic acid, pyochelin, 15-hydroxy-5Z,8Z,11Z,13E-eicosatetraenoic acid, (Z)-7-[(2R,3S)-3-[(2Z,5E)-Undeca-2,5-dienyl]oxiran-2-yl]hept-5-enoic acid, arginylasparagine, cholic acid, sphinganine, elaidic acid, gossypin, L-carnosine, tetrodotoxin, and ursodiol.

Both qualitative and quantitative tests were conducted to investigate plant growth-promoting traits of the isolated strain *P. polymyxa* SK1. These tests included the detection of organic acids, indole acetic acid (IAA), ACC deaminase, siderophores, nitrogen fixation, and phosphate solubilization.

**Figure 5:**
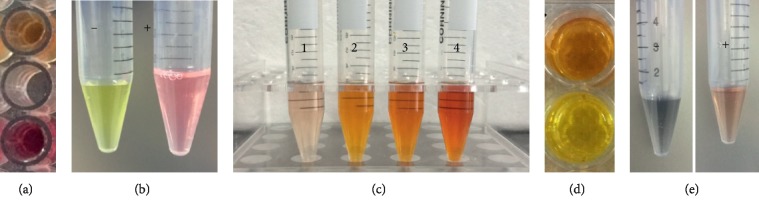
Qualitative detection of plant growth-promoting traits in the isolated *P. polymyxa* SK1. (a) Organic acid production was revealed by a color change to yellow in the upper well, while extreme lower well with pink color was used as a negative control. (b) Qualitative detection of IAA showing a change of coloration from yellow to pink. (c) IAA detection supplemented with various tryptophan concentrations. The tubes numbered with 1, 2, 3, and 4 show IAA detection at 0 mg·ml^−1^, 2 mg·ml^−1^, 4 mg·ml^−1^, and 6 mg·ml^−1^ tryptophan concentrations, respectively. (d) ACC deaminase activity. The upper well with brown coloration showed ACC deaminase detection while the lower well with yellow color was used as a negative control. (e) Production of siderophores was confirmed by a change of color from blue (−) to yellow/orange (+) as indicated by CAS assay.

The ability of the isolated strain for the production of organic acid was assayed qualitatively. Strain SK1 showed positive results for organic acid production (Table 3 and Figure 5(a)). Organic acids were detected qualitatively as a change of color from pink to yellow.

Both qualitative and quantitative assays were conducted for indole acetic acid (IAA) production by the isolate. The qualitative test confirmed IAA production in the SK1 isolate through the change of color of the culture supernatant from yellow to pink (Figure 5(b)). IAA was quantified in the strain at various tryptophan concentrations supplemented in the culture medium. The strain was able to produce IAA at different tryptophan concentrations (Table 3 and Figure 5(c)). The IAA contents in the isolate increased with increasing tryptophan in the culture medium. The strain SK1 showed lower IAA content, i.e., 15.17 ± 0.9 *μ*g ml^−1^, at 0 mg·ml^−1^ tryptophan concentration. The IAA contents increased gradually with the increase in the tryptophan concentrations from 0 mg·ml^−1^ to 6 mg·ml^−1^. The IAA contents produced by SK1 at tryptophan concentrations of 2 mg·ml^−1^, 4 mg·ml^−1^, and 6 mg·ml^−1^ in the culture medium were recorded as 52.67 ± 2.6 mg·ml^−1^, 79.50 ± 4.6 mg·ml^−1^, and 109.67 ± 5.8 mg·ml^−1^, respectively. These results suggest that the isolated SK1 strain is able to produce high content of IAA in the culture medium. Exogenous tryptophan had no negative impact on IAA accumulation; rather, a positive correlation was found between tryptophan and IAA.

The potential of the isolated *P. polymyxa* SK1 to produce 1-aminocyclopropane-1-carboxylate (ACC) deaminase was analyzed through a qualitative test. The strain was found positive for the production of ACC deaminase as revealed by a change of color from yellow to brown (Figure 5(d)). The strength of the (ACC) deaminase activity was found as moderate (Table 3).

**Figure 6:**
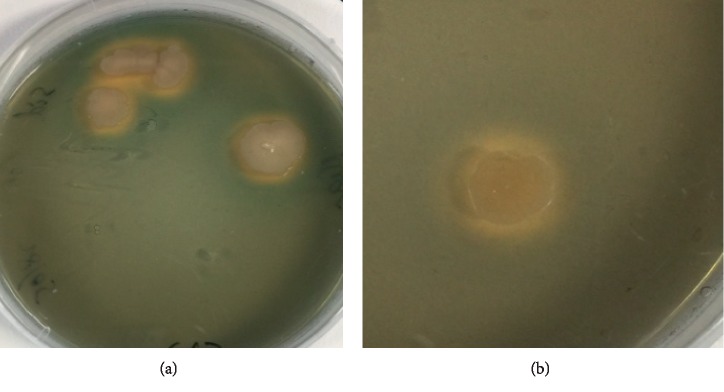
Qualitative analysis of siderophores on CAS blue agar plates. Siderophores in *P. polymyxa* SK1 were detected as a yellow/orange hallow surrounding the bacterial colonies (a). A closer look at the surrounding hallow (b).

Siderophores production in the isolate was assayed at different Fe(III) citrate concentrations supplied in the culture medium. The qualitative test confirmed siderophore production through a change of color from blue to orange-yellow (Figure 5(e)). Further quantification of siderophores in the isolate was conducted at various Fe(III) citrate concentrations (Table 3). Higher siderophores were detected when the isolate was cultured in medium without Fe(III) citrate. Strain SK1 showed 41.23 ± 3.4 (psu) in culture medium without the addition of Fe(III) citrate. The siderophores accumulation decreased with the addition of Fe(III) citrate in the medium. A slight decrease in siderophores was noticed at 0.25 *μ*M Fe(III) citrate in the medium where 36.32 ± 1.4 (psu) was accumulated. However, a significant reduction in siderophores was observed when Fe(III) citrate concentration was raised to 3.0 *μ*M. At this Fe(III) citrate concentration, the strain SK1 showed 18.23 ± 0.9 (psu) siderophores. The production of siderophores was further confirmed through a qualitative test using chrome azurol S (CAS) assay on agar plates. The SK1 strain showed an orange/yellow hallow surrounding the individual colonies as an indication of siderophore production and quenching of iron from the dye complex (Figures 6(a) and 6(b). Moreover, the diameter of the yellow/orange hallow around SK1 colony averaged 7.2 ± 0.45 mm. This result further confirmed that the endophytic *P. polymyxa* SK1 strain is capable of producing siderophores.

**Figure 7:**
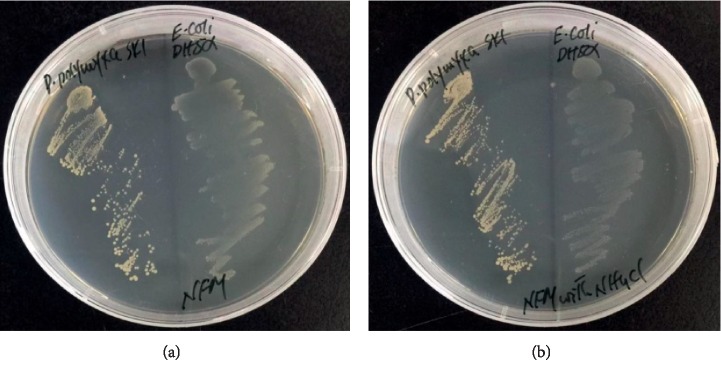
N_2_-fixation by the endophytic *P. polymyxa* SK1. The isolated strain was inoculated on nitrogen-deficient malate medium (NFM) and was observed for growth in reference to nonnitrogen fixing *E. coli* DH5α. Growth of both strains on NFM medium (a) and NFM medium supplemented with 5 mM NH_4_Cl (b).

The *P. polymyxa* strain SK1 was assessed for its potential to fix atmospheric nitrogen. This was done by the ability of the strain to grow on nitrogen-free minimal medium (NFM). In this experiment, the *Escherichia coli* strain DH5*α*, which is unable to grow on nitrogen-free medium, was used as a negative control. Both SK1 and *E. coli* strains were cultured on NFM. The same medium supplemented with 5 mM NH_4_Cl, a preferred source of nitrogen, was used to check that both strains could grow on it. Our results showed a clear difference in the growth pattern of both strains on the NFM medium. Endophytic *P. polymyxa* SK1 was able to grow on nitrogen-free medium while *E. coli* DH5*α*, which does not fix nitrogen, failed to grow on it (Figure 7(a)). The *E. coli* strain grew only on medium supplemented with reactive nitrogen (Figure 7(b)). These results clearly indicated the ability of the isolated *P. polymyxa* SK1 to fix atmospheric nitrogen.

**Figure 8:**
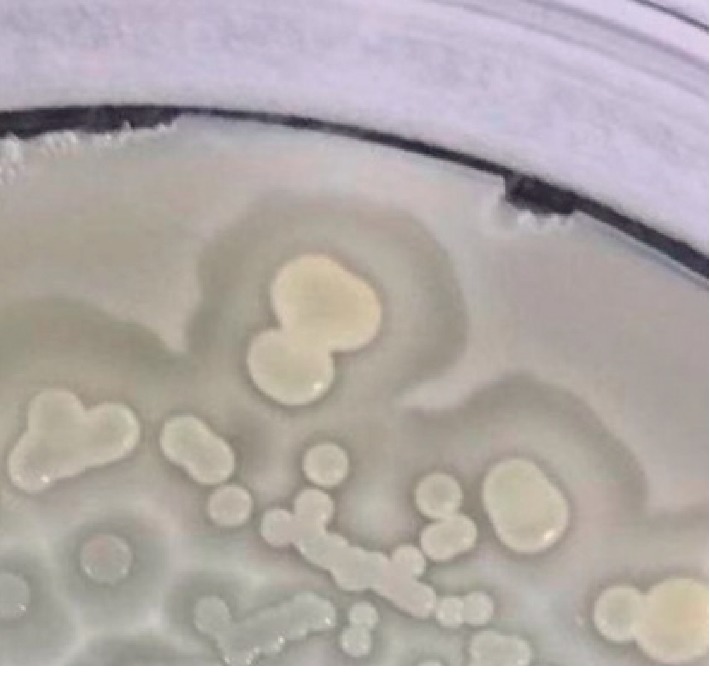
Phosphate solubilization assay of *P. polymyxa* SK1. Phosphate solubilization activity of SK1 was assayed on the NBRIP medium as a clearing area surrounding bacterial colonies.

The ability of the isolated strain *P. polymyxa* SK1 to solubilize inorganic phosphate was assayed on solid NBRIP medium. This medium was supplemented with Ca_3_(PO_4_)_2_ as the sole source of inorganic phosphate. The endophytic strain SK1 was able to grow on the medium for longer incubation time and it produced hallows around the individual colonies (Figure 8). This was a clear indication of the ability of the SK1 strain to utilize inorganic phosphate in the medium.

**Figure 9:**
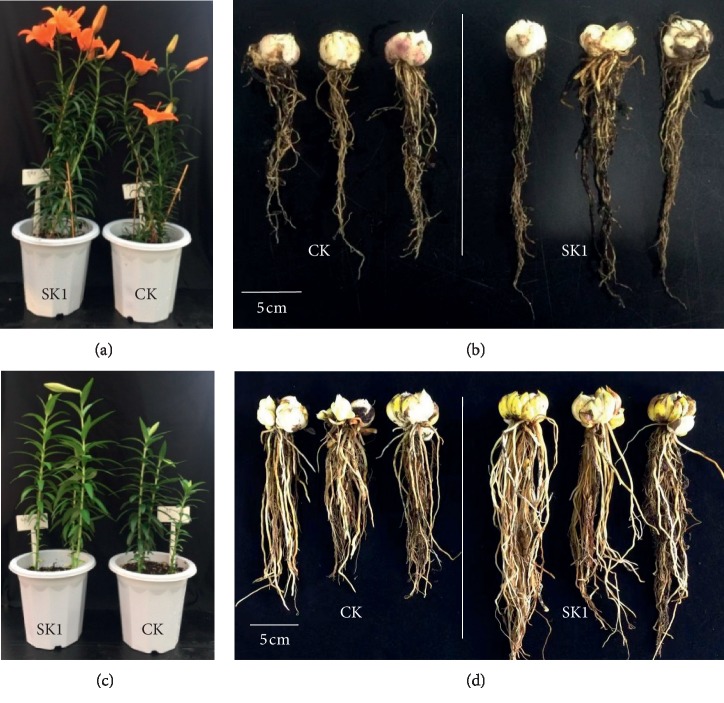
Plant growth promotion in *Lilium* varieties upon *P. polymyxa* SK1 inoculation. SK1-inoculated and noninoculated control (CK) plants of Tresor variety showed clear differences for plant height (a) and bulbs and root morphology (b). Similar differences were found for plant height and bulbs and root morphology between inoculated and control plants of White Heaven variety (c) and (d).

The plant growth-promoting effects of the *P. polymyxa* SK1 were investigated on the growth and productivity of two *Lilium* varieties grown from bulbs under greenhouse conditions. The bulbs of the two commercially cultivated varieties, the Asiatic Hybrid “Tresor” and “White Heaven,” were inoculated with the SK1 endophytic isolate. Upon completion of the vegetative growth of both varieties, several morphological growth parameters were measured. These included plant height, number of flowering shoots, leaf length, leaf width, stem diameter, weight of bulbs, and root length. Improvement of vegetative growth was observed in both varieties (Tables 4 and 5). The Tresor variety showed a positive response to bacterial inoculation in terms of improved growth parameters (Table 4). Some growth parameters such as plant height, leaf length, and root length were found significantly higher than those of the noninoculated control plants. The inoculated plants of Tresor variety showed significantly higher (*P* ≤ 0.05) plant height (55.1 ± 5.0 cm) as compared to that of noninoculated control plants which showed 47.8 ± 10.2 cm plant height (Figure 9(a)). Leaf length was also found significantly different between the inoculated and control plants of Tresor variety. Inoculated plants showed significantly higher (*P* ≤ 0.05) leaf length, i.e., 94.3 ± 8.6 mm, as compared to 89.2 ± 11.3 mm in noninoculated control plants. Moreover, the SK1-inoculated plants of “Tresor” produced significantly longer roots than the control plants (Figure 9(b)). Inoculated plants showed a root length of 27.4 ± 3.1 mm as compared to 17.0 ± 3.8 mm of control plants. Other growth parameters such as the number of flowering shoots, leaf width, stem diameter, and bulbs weight were also found higher in inoculated plants than those in the control plants. However, these differences were nonsignificant. Flowers production is one of the required traits in *Lilium* plants due to its commercial value in the cut flower industry. In the present experiment, the Tresor variety exhibited more flowers production than that of the control plants. Although the differences were not significant, some inoculated plants produced more than three flowers, as compared to the average two and three flowers in the noninoculated control plants. The White Heaven variety also showed better vegetative growth upon SK1 inoculation (Table 5). This variety generally produces one flower per plant. This character was found unchanged in both inoculated and uninoculated plants. Differences were found in other growth parameters such as plant height, leaf length and width, stem diameter, bulbs weight, and root length. Plant height as an important growth parameter was increased significantly in inoculated plants, which was found 40.2 ± 9.2 cm as compared to 31.2 ± 8.7 cm in noninoculated control plants (Table 5 and Figure 9(c)). Leaf width was also found significantly different in both inoculated and control plants. More importantly, the inoculated plants produced bulbs with significantly higher (*P* ≤ 0.05) weight and longer roots (Figure 9(d)). Inoculated plants exhibited an average bulb weight of 22.7 ± 3.6 g relative to 15.9 ± 3.9 g of the control plants. Inoculated plants exhibited 23.2 ± 3.4 cm root length as compared to 17.1 ± 5.6 of control plants. As a whole, both varieties showed growth improvement as a result of inoculation with the isolated SK1 strain.

## 4. Discussion

A new bacterial strain SK1 of *Paenibacillus polymyxa* was isolated from the bulb parts of *Lilium lancifolium*. The isolated strain showed antimicrobial activity and resisted the growth and proliferation of important bacterial pathogens. The identity of the isolate was determined after morphological and molecular analysis. Further tests confirmed that the strain is involved in plant growth promotion and acts as a potential biocontrol agent against phytopathogens that cause severe diseases in various crop plants. In previous studies, strains of *P. polymyxa* have been shown to protect cauliflower, pea, ginseng, cucumber, chickpea, peanut, soybean, and pepper against phytopathogens [11]. In the present study, the isolated strain exhibited variable antimicrobial/antiproliferative effects against fungal phytopathogens like *Fusarium oxysporum*, *Botryosphaeria dothidea*, *Botrytis cinerea*, and *Fusarium fujikuroi*. These phytopathogens may cause diseases in *Lilium* species as revealed by a test confirming pathogenicity potential against cultivated species/varieties like *Lilium davidii* and Tresor. One of these phytopathogens, *Fusarium fujikuroi*, was isolated and identified from *in vitro* bulbs of *Lilium wardii*, a *Lilium* species. This could be a disease-causing agent in *Lilium wardii* and other *Lilium* species. Interestingly, the isolated endophytic strain SK1 exhibited higher antifungal activities against all tested fungal pathogens and was found very effective against the growth and proliferation of *Fusarium fujikuroi*. Further tests would be required to confirm the disease-causing potential of *Fusarium fujikuroi* in *Lilium* species and the protection conferred by the isolated strain.

Many species of *Paenibacillus* have been reported to produce antimicrobial compounds, chemicals, and enzymes with potential uses in medicine, pesticides, and bioremediation [11]. The broad-spectrum antagonistic activity of several strains of *P. polymyxa* has been attributed to their ability to produce antimicrobial compounds, which effectively prevented various plant diseases caused by bacteria, fungi, and nematodes [12]. *P. polymyxa* strains were reported to produce two types of peptide antibiotics, such as polypeptins, polymyxins, gavaserin, jolipeptin, and saltavalin against bacteria and several LI-F antibiotics, including fusaricidins and gatavalin against fungi, and actinomycetes [36, 37]. In the present study, the ethyl acetate fraction showed a list of putative compounds including antimicrobial compounds like dipeptides, tripeptides, cyclopeptides (cyclo-(Leu-Leu), cyclo(Pro-Phe)), 2-heptyl-3-hydroxy 4-quinolone, 6-oxocativic acid, anhydrobrazilic acid, 1-(5-methoxy-1H-indol-3-yl)-2-piperidin-1-ylethane-1,2-dione, 5-hydroxy-2-[2-hydroxy-3-[(2S,3R,4S,5S,6R)-3,4,5-trihydroxy-6-(hydroxymethyl)oxan-2-yl]oxyphenyl]-7,8-dimethoxychromen-4-one, Octadecenoic acid, 15-hydroxy-(5Z,8Z,11Z,13E)-eicosatetraenoic acid, pyochelin, (Z)-7-[(2R,3S)-3-[(2Z,5E)-undeca-2,5-dienyl]oxiran-2-yl]hept-5-enoic acid, cholic acid, ursodiol, elaidic acid, gossypin, sphinganine, and tetrodotoxin. Previous studies reported these compounds from various endophytes where they showed antimicrobial effects. The presence of diketopiperazines (cyclodipeptides) in *Pseudomonas* and *Bacillus* species resulted in broad-spectrum antimicrobial activities [38, 39]. *Lactobacillus plantarum* AF1 isolated from kimchi showed the presence of cyclo(Leu-Leu) that exhibited antifungal activity against *Aspergillus flavus* [40]. Cyclo (Pro-Phe) identified from *Bacillus amyloliquefaciens* Q-426 showed significant antifungal activity [41]. In addition, dipeptides and tripeptides have also been proved effective in conferring fungal resistance [42]. The 2-heptyl-3-hydroxy 4-quinolone was reported to possess antimicrobial activities and acts as an iron chelator [43]. In *Pseudomonas*, this compound provided the core for the quinolone-based QS system called *Pseudomonas* quinolone signal (PQS). In addition to its role as a QS signaling molecule, the PQS was reported to mediate iron acquisition, cytotoxicity, and resistance to environmental stresses including infectious disease-causing agents [44, 45]. Oxocativic acid is a diterpene that was previously reported from *Cistus ladanifer* L. with a potential role in the allelopathic activity of this Mediterranean species [46]. Similarly, octadecenoic acid from various hosts exhibited antimicrobial properties [47]. The compound 15-hydroxy-(5Z,8Z,11Z,13E)-eicosatetraenoic acid putatively identified in the present study was previously reported along with a number of other compounds from Sea Slater *Ligia exotica*, a source of Chinese folk medicine with pain-relieving and anti-inflammatory properties [48]. Pyochelin is basically a siderophore produced by endophytes and was previously known only as an iron chelator. However, it is established now that it may also play a potential role in plant resistance to pathogens [49]. Likewise, the putative detection of pyochelin in the present study might have a possible role in siderophores accumulation and antifungal resistance of the *P. polymyxa*, SK1. The other compounds identified in the SK1 were cholic acid, ursodiol, and elaidic acid. These compounds were previously reported as antimicrobial in nature [50–52]. Gossypin was previously reported as having antimicrobial properties [53]. Sphinganine belonging to sphingosines and sphingolipids was identified in the isolated strain SK1. Previous studies have demonstrated that several sphingoid bases and fatty acids act as antimicrobial agents [54, 55]. Tetrodotoxin (TTX) is a strong marine toxin which is a powerful sodium channel inhibitor. Alabssawy [56] demonstrated the antimicrobial activity of tetrodotoxin isolated from the puffer fish *Lagocephalus sceleratus*. The presence of these putative compounds in the isolated *P. polymyxa* SK1 strain might have contributed to the high antagonistic effects against the tested fungal pathogens.

Previous studies have revealed that the plant growth-promoting traits in isolated strains of *P. polymyxa* were correlated with several mechanisms such as the production of organic acids, siderophores, lowering plant ethylene levels by ACC deaminase production, synthesis of plant growth-regulating hormones like indole acetic acid and cytokinins, nitrogen fixation, and phosphate solubilization [3, 36]. In the present study, we confirmed through various tests that the isolated strain SK1 possessed plant growth-promoting traits. SK1 was capable of producing organic acids as revealed by a qualitative test. Organic acids play an important role in plant growth promotion and defense against phytopathogens [57, 58]. *Paenibacillus*-produced organic acids, such as oxalic acid, may also contribute to iron uptake [59].

IAA is an important auxin that directly promotes plant growth and development. Plants can produce their own phytohormones but may also utilize foreign sources such as those produced by microorganisms. Other than plants, IAA is synthesized by plant-associated bacteria and fungi, including *Paenibacillus* [60]. The ability of *Paenibacillus* to produce IAA is considered as a major contributor to plant growth promotion [61]. In this connection, the role of the tryptophan precursor of IAA is important as considerable amounts of IAA are produced in the presence of excess tryptophan [61]. In the present study, the exogenous tryptophan concentrations had no negative impact on IAA production in SK1 and a positive correlation was observed between the tryptophan concentrations and IAA production. Some previous studies also showed a positive correlation between the exogenous application of tryptophan and the IAA production in the isolated rhizosphere microbes and endophytes [62, 63]. Sasirekha et al. [64] reported induction in IAA production by the isolated strain, *Pseudomonas aeruginosa.* Tryptophan supplementation (0.1 g·L^−1^) induced a 5-fold increase in IAA production (80 *μ*g·mL^−1^) when compared to control (without tryptophan). Other studies conducted in isolated strains of *Serratia plymuthica* and *Paenibacillus polymyxa* also reported tryptophan mediated induction of IAA production [65, 66]. It seems that the isolated strain SK1 produced IAA in a tryptophan-dependent pathway. In addition, some recent studies reported growth-promoting traits including IAA production in *Paenibacillus* strains isolated from various hosts. Xu and Kim [67] isolated *Paenibacillus* strains from the soil and one strain, *P. polymyxa* SC09-21, resulted in plant growth promotion and suppression of Fusarium crown and root rot. The *P. polymyxa* strain CR1 isolated from degrading corn roots produced IAA and showed antagonistic activities against common plant pathogens and improved growth of maize, potato, cucumber, *Arabidopsis*, and tomato plants upon inoculation [12].

In this study, the isolated *P. polymyxa* SK1 strain exhibited ACC deaminase activity as revealed by a qualitative test. Production of 1-aminocyclopropane-1-carboxylate (ACC) deaminase is one of the important characteristics of plant growth-promoting microbes and endophytes. ACC deaminase cleaves ACC, the immediate precursor of the plant hormone ethylene, to produce *α*-ketobutyrate and ammonia [68]. Ethylene serves as an important signaling molecule in plants under biotic and abiotic stresses and results in plant growth inhibition [69]. Previous studies have reported that inoculation of plants with ACC deaminase producing microbes decreased ethylene levels that resulted in a decrease in the inhibition of plant growth under biotic and abiotic stresses [4, 70]. Our results are supported by previous reports of ACC deaminase production in several isolated strains of *Paenibacillus* and *Bacillus* [71–73].

Iron is abundant in the soil as a nonbioavailable form of largely insoluble Fe^3+^ oxyhydroxides and cannot be used by plants and microorganisms. Plant-associated microbes reduce Fe^3+^ to Fe^2+^ with the help of ferrireductases or solubilize it with extracellular Fe^3+^ chelators called siderophores, which are released under limited iron in the soil [59]. Both plants and microbes have access to these soluble Fe^3+^-siderophore complexes [74]. Previous studies have shown siderophores production in the isolated PGPRs including species of *Paenibacillus* [75, 76]. Raza and Shen et al. [59] reported siderophores production in the isolated strain *Paenibacillus polymyxa* SQR-21 under different iron conditions. In the present study, the isolated strain SK1 produced siderophores, which were recorded by both qualitative and quantitative methods. Quantitative evaluation of siderophores was conducted under different iron conditions. Siderophores production was maximum when there was no Fe(III) citrate in the medium. However, increasing iron concentrations in the medium negatively impacted the siderophores production. These results are in complete agreement with previous reports where the inverse relationship was observed between different iron concentrations and siderophores production [77, 78].

Nitrogen is an essential and vital element for the normal growth and development of plants. Several plant growth-promoting bacteria including species of *Paenibacillus* were found with the ability to fix atmospheric nitrogen and this trait contributed to the positive effects on target plants upon inoculation of the endophytes in some examples. Anand et al. [10] reported growth promotion in lodgepole pine inoculated with *P. polymyxa* P2b-2R in N_2_-limited soil. They concluded that the improved growth was due to the N_2_-fixation ability of *P. polymyxa*. Furthermore, Puri et al. [79] demonstrated that inoculation of canola (*Brassica napus* L.) with the same strain *P. polymyxa* P2b-2R resulted in nitrogen fixation and growth promotion. The isolated strain SK1 was also able to solubilize inorganic phosphate as revealed from the results. Previous reports have confirmed that phosphate solubilization is one of the important traits of *P. polymyxa* strains to promote the growth of associated plants. Wang et al. [80] demonstrated the phosphate solubilizing ability of *Paenibacillus* strains from mycorrhizal and nonmycorrhizal cucumber plants. More recently, Mohd Din et al. [81] reported phosphate solubilization activity of *P. polymyxa* under abiotic stress condition and its role in promoting the growth of maize seeds. This shows that *P. polymyxa* strains are ideal candidates to promote sustainable agriculture as a result of enhanced soil fertility and disease resistance.

In the present study, inoculation of two *Lilium* varieties, Tresor and White Heaven, with *P. polymyxa* SK1 resulted in growth improvement than the noninoculated control plants. Moreover, the inoculated plants of both genotypes exhibited improved bulbs production and longer roots. Particularly, the roots were comparatively longer in the inoculated plants, though all plants were irrigated normally without any drought stress. However, underwater scarcity or drought conditions, the longer roots in the SK1-inoculated plants may provide a selective advantage by providing access to more water as compared to that of control plants. This possibility could be tested as a future strategy. Overall, our results suggest that *P. polymyxa* SK1 harbors multiple plant growth-promoting traits which resulted in enhanced plant growth of *Lilium* varieties.

## 5. Conclusion

The endophytic *Paenibacillus polymyxa* SK1, isolated from *Lilium lancifolium*, exhibited antagonistic effects against agriculturally important plant pathogens. The antifungal nature of the isolated strain was confirmed through putative identification of a number of bioactive secondary metabolites with reported antimicrobial effects. The strain SK1 showed multiple plant growth-promoting effects that resulted in vegetative and reproductive growth improvement of *Lilium* varieties. Owing to these beneficial traits, the endophytic *P. polymyxa* SK1 may play an important role in soil fertility, plant growth promotion, and disease control.

## Figures and Tables

**Table 1:** Percent zones of inhibition of the test pathogens on PDA media inoculated with the endophytic bacterial isolates.

Bacterial isolate	Tested fungal pathogens			
	*Fusarium oxysporum*	*Botryosphaeria dothidea*	*Botrytis cinerea*	*Fusarium fujikuroi*
SK1	55.54 ± 2.89%	66.67 ± 2.23%	61.19 ± 3.12%	60.71 ± 3.53%

Means are averages ± standard deviation (SD) of three replicates with *n* = 5.

**Table 2:** Overview of the putative secondary metabolites detected in the ethyl acetate fraction of *P. polymyxa* SK1.

No.	Compound name	*m/z* measured	Library *m/z*	Molecular formula	Adduct	GNPS score	GNPS library ID	CAS no.
1	2-heptyl-3-hydroxy 4-quinolone	260.327	260	C_16_H_21_NO_2_	M + H	0.923445	CCMSLIB00000006838	108985-27-9
2	6-Oxocativic acid	320.115	321.242	C_20_H_32_O_3_	[M + H]^+^	0.776846	CCMSLIB00004717850	N/A
3	Anhydrobrazilic acid	236.599	235.06	C_12_H_10_O_5_	[M + H]^+^	0.801906	CCMSLIB00004716915	N/A
4	Cer(d18 : 1/0 : 0)	301.257	299.282	C_18_H_38_N_1_O_2_	M + H	0.80382	CCMSLIB00003119317	N/A
5	cyclo-(Leu-Leu)	226.649	227.174	C_12_H_22_N_2_O_2_	M + H	0.786044	CCMSLIB00000081186	
6	Cyclo(Pro-Phe)	246.002	245.01	C_14_H_16_N_2_O_2_	M + H	0.721089	CCMSLIB00003134825	511126
7	1-(5-Methoxy-1H-indol-3-yl)-2-piperidin-1-ylethane-1,2-dione	286.183	287.14	C_16_H_18_N_2_O_3_	M + H	0.762453	CCMSLIB00000079850	N/A
8	5-hydroxy-2-[2-hydroxy-3-[(2S,3R,4S,5S,6R)-3,4,5-trihydroxy-6-(hydroxymethyl)oxan-2-yl]oxyphenyl]-7,8-dimethoxychromen-4-one	494.59	493.134	C_23_H_24_O_12_	M + H	0.700858	CCMSLIB00000846731	N/A
9	Octadecenoic acid	282.424	283.264	C_18_H_34_O_2_	M + H	0.786834	CCMSLIB00000075360	
10	Pyochelin	325.953	325	C_14_H_16_N_2_O_3_S_2_	M + H	0.911325	CCMSLIB00000006841	69772-54-9
11	15-hydroxy-5Z,8Z,11Z,13E-eicosatetraenoic acid	317.725	319.229	C_20_H_32_O_3_	M-H	0.723226	CCMSLIB00003140178	73836870
12	(Z)-7-[(2R,3S)-3-[(2Z,5E)-Undeca-2,5-dienyl]oxiran-2-yl]hept-5-enoic acid	304.244	303.232	C_20_H_32_O_3_	M + H-H_2_O	0.767871	CCMSLIB00003136522	1.84E+08
13	Arginylasparagine	290.936	289.15	C_10_H_20_N_6_O_4_	M + H	0.755764	CCMSLIB00003136310	N/A
14	Cholic acid	372.736	373.26	C_24_H_40_O_5_	M + H − 2H_2_O	0.749273	CCMSLIB00003135877	81254
15	Sphinganine	303.453	302.303	C_18_H_39_NO_2_	M + H	0.798672	CCMSLIB00003137456	764227
16	Elaidic acid	283.702	283.264	C_18_H_34_O_2_	M + H	0.716292	CCMSLIB00003138006	112798
17	Gossypin	482.298	481.1	C_21_H_20_O_13_	M + H	0.796452	CCMSLIB00003137147	652788
18	His-Pro	235.256	235.12	C_11_H_16_N_4_O_3_	M + H − H_2_O	0.902967	CCMSLIB00003139663	N/A
19	Ile-Pro	211.99	211.144	C_11_H_20_N_2_O_3_	M + H − H_2_O	0.897641	CCMSLIB00003139619	N/A
20	Ile-Pro-Ile	342.436	342.239	C_17_H_31_N_3_O_4_	M + H	0.873283	CCMSLIB00003139778	90614485
21	L-Carnosine	225.163	227.11	C_9_H_14_N_4_O_3_	M + H	0.780065	CCMSLIB00003134824	305840
22	Leu-His	251.092	251.15	C_12_H_20_N_4_O_3_	M + H − H_2_O	0.926839	CCMSLIB00003138111	N/A
23	Leu-Pro	228.618	229.16	C_11_H_20_N_2_O_3_	M + H	0.930163	CCMSLIB00003139840	N/A
24	Maltulose	364.32	365.11	C_12_H_24_O_12_	M + Na	0.737391	CCMSLIB00003135808	17606723
25	Phe-Pro	263.036	263.14	C_14_H_18_N_2_O_3_	M + H	0.980382	CCMSLIB00003139670	N/A
26	Phe-Pro-Lys	196.143	196.119	C_20_H_30_N_4_O_4_	[M + 2H]	0.797602	CCMSLIB00003137655	N/A
27	Val-Phe	245.168	247.14	C_14_H_20_N_2_O_3_	M + H − H_2_O	0.920362	CCMSLIB00003135756	N/A
28	Tetrodotoxin	318.932	320.109	C_11_H_17_N_3_O_8_	M + H	0.779742	CCMSLIB00003740012	4368-28-9
29	Ursodiol	376.622	375.289	C_24_H_40_O_4_	M + H	0.723467	CCMSLIB00000005521	N/A

**Table 3:** Plant growth-promoting (PGP) traits of the isolated *P. polymyxa* SK1.

OA	IAA (*μ*g/ml) at different tryptophan concentrations				ACC	Siderophores (psu) at different Fe(III) citrate concentrations			NA	PS
	0 mg ml^−1^	2 mg ml^−1^	4 mg ml^−1^	6 mg ml^−1^		0 *μ*M	0.25 *μ*M	3.0 *μ*M		
**+++**	15.17 ± 0.9	52.67 ± 2.6	79.50 ± 4.6	109.67 ± 5.8	**++**	41.23 ± 3.4	36.32 ± 1.4	18.23 ± 0.9	++	+

Note: results are means ± standard deviation of three independent experiments with each treatment measured three times. Organic acids (OA), ACC deaminase (ACC), Indole acetic acid (IAA), Nitrogenase activity (NA), and Phosphate solubilization (PS). Evaluation of the positivity to the tests: negative (−) shows the absence of activity, while (+) shows lower to the highest activity (+++).

**Table 4:** Analysis of growth parameters of Tresor variety upon inoculation of *P. polymyxa* SK1.

Treatments	# of flowering shoots/plant	Plant height (cm)	Leaf length (mm)	Leaf width (mm)	Stem diameter (mm)	Bulbs weight (g)	Root length (cm)
CK	2.6 ± 0.5^a^	47.8 ± 10.2^a^	89.2 ± 11.3^a^	9.7 ± 1.4^a^	8.9 ± 0.7^a^	12.9 ± 1.4^a^	17.0 ± 3.8^a^
SK1	3.0 ± 0.5^a^	55.1 ± 5.0^b^	94.3 ± 8.6^b^	11.1 ± 1.1^a^	9.5 ± 1.0^a^	14.3 ± 2.1^a^	27.4 ± 3.1^b^

Means are averages ± standard deviations (SD). Values in a column with different letters are significantly different by Student's *t*-test at (*P* ≤ 0.05).

**Table 5:** Analysis of growth parameters of White Heaven variety upon inoculation with *P. polymyxa* SK1.

Treatments	Plant height (cm)	Leaf length (mm)	Leaf width (mm)	Stem diameter (mm)	Bulbs weight (g)	Root length (cm)
CK	31.2 ± 8.7^a^	97.6 ± 12.3^a^	16.9 ± 2.2^a^	7.9 ± 0.9^a^	15.9 ± 3.9^a^	17.1 ± 5.6^a^
SK1	40.2 ± 9.2^b^	99.8 ± 13.6^a^	19.4 ± 2.1^b^	8.4 ± 1.3^a^	22.7 ± 3.60^b^	23.2 ± 3.4^b^

Means are averages ± standard deviations (SD). Values in a column with different letters are significantly different by Student's t-test at (*P* ≤ 0.05).

## Data Availability

The data used to support the findings of this study are available from the corresponding author upon request.

## Supplementary Materials

Supplementary Materials Figure S1: standard curve based on known quantities of indole acetic acid (IAA). Each dot represents average reading of three replicates. Figure S2: *in vitro* pathogenicity test of the fungal pathogens against Asiatic Hybrid “Tresor” and *Lilium davidii*. Bulbs of Tresor and *L. davidii* were inoculated with fungal pathogens through putting mycelia on the wounded surface of the bulbs. Plate (A) is the control with bulbs of Tresor without fungal inoculation, while plates (B), (C), (D), and (E) show bulbs inoculation with *Botryosphaeria dothidea*, *Fusarium fujikuroi*, *Fusarium oxysporum*, and *Botrytis cinerea*, respectively. Plate (F) is the control of *L. davidii* without fungal inoculation, while plates (F), (G), (H), and (I) show bulbs inoculated with *Botryosphaeria dothidea*, *Fusarium fujikuroi*, *Fusarium oxysporum*, and *Botrytis cinerea*, respectively.
