# Supplementary material for: Isolation and Characterization of Plant Growth-Promoting Endophytic Bacteria Paenibacillus polymyxa SK1 from Lilium lancifolium
Source: Biomed Res Int. 2020 Feb 27;2020:8650957. doi: 10.1155/2020/8650957 (PMC7064867; doi:10.1155/2020/8650957)
Supplement: Supplementary Materials — Figure S1: standard curve based on known quantities of indole acetic acid (IAA). Each dot represents average reading of three replicates. Figure S2: in vitro pathogenicity test of the fungal pathogens against Asiatic Hybrid “Tresor” and Lilium davidii. Bulbs of Tresor and L. davidii were inoculated with fungal pathogens through putting mycelia on the wounded surface of the bulbs. Plate (A) is the control with bulbs of Tresor without fungal inoculation, while plates (B), (C), (D), and (E) show bulbs inoculation with Botryosphaeria dothidea, Fusarium fujikuroi, Fusarium oxysporum, and Botrytis cinerea, respectively. Plate (F) is the control of L. davidii without fungal inoculation, while plates (F), (G), (H), and (I) show bulbs inoculated with Botryosphaeria dothidea, Fusarium fujikuroi, Fusarium oxysporum, and Botrytis cinerea, respectively. [file 8650957.f1.docx]

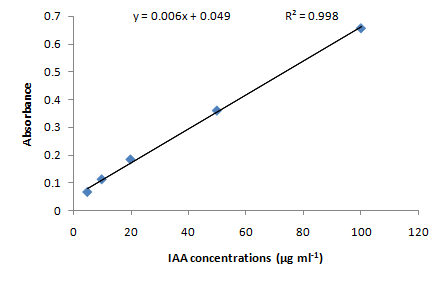


Figure S1: Standard curve based on known quantities of indole acetic acid (IAA). Each dot represents average reading of three replicates.


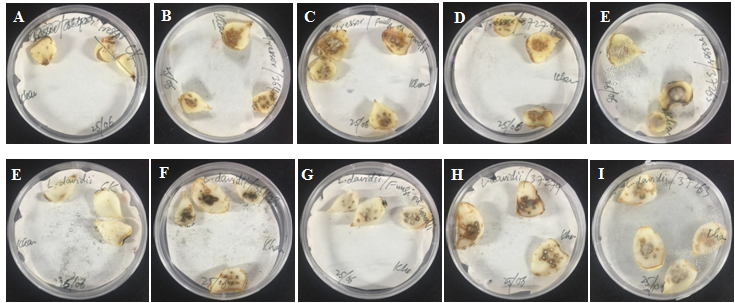


Figure S2: In vitro pathogenicity test of the fungal pathogens against Asiatic Hybrid "Tresor" and Lilium davidii. Bulbs of Tresor and L. davidii were inoculated with fungal pathogens through putting mycelia on the wounded surface of the bulbs. Plate (A) is the control with bulbs of Tresor without fungal inoculation. While plates (B), (C), (D), and (E) show bulbs inoculation with Botryosphaeria dothidea, Fusarium fujikuroi, Fusarium oxysporum, and Botrytis cinerea, respectively. Plate (F) is the control of L. davidii without fungal inoculation, while plates (F), (G), (H) and (I) show bulbs inoculated with Botryosphaeria dothidea, Fusarium fujikuroi, Fusarium oxysporum, and Botrytis cinerea, respectively
